# Intrinsic Cortico-Subcortical Functional Connectivity in Developmental Dyslexia and Developmental Coordination Disorder

**DOI:** 10.1093/texcom/tgaa011

**Published:** 2020-04-06

**Authors:** Fabien Cignetti, Federico Nemmi, Marianne Vaugoyeau, Nadine Girard, Jean-Michel Albaret, Yves Chaix, Patrice Péran, Christine Assaiante

**Affiliations:** 1 University of Grenoble Alpes, CNRS, TIMC-IMAG, F-38000 Grenoble, France; 2 ToNIC, Toulouse NeuroImaging Center, Université de Toulouse, Inserm, UPS, 31024 Toulouse, France; 3 Aix Marseille University, CNRS, LNC, 13331 Marseille, France; 4 Aix Marseille University, CNRS, Fédération 3C, 13331 Marseille, France; 5 Aix Marseille University, CNRS, CRMBM, 13385 Marseille, France

**Keywords:** cortico-subcortical networks, developmental coordination disorder, developmental dyslexia, machine learning, resting-state fMRI

## Abstract

Developmental dyslexia (DD) and developmental coordination disorder (DCD) are distinct diagnostic disorders. However, they also frequently co-occur and may share a common etiology. It was proposed conceptually a neural network framework that explains differences and commonalities between DD and DCD through impairments of distinct or intertwined cortico-subcortical connectivity pathways. The present study addressed this issue by exploring intrinsic cortico-striatal and cortico-cerebellar functional connectivity in a large (*n* = 136) resting-state fMRI cohort study of 8–12-year-old children with typical development and with DD and/or DCD. We delineated a set of cortico-subcortical functional circuits believed to be associated with the brain’s main functions (visual, somatomotor, dorsal attention, ventral attention, limbic, frontoparietal control, and default-mode). Next, we assessed, using general linear and multiple kernel models, whether and which circuits distinguished between the groups. Findings revealed that somatomotor cortico-cerebellar and frontoparietal cortico-striatal circuits are affected in the presence of DCD, including abnormalities in cortico-cerebellar connections targeting motor-related regions and cortico-striatal connections mapping onto posterior parietal cortex. Thus, DCD but not DD may be considered as an impairment of cortico-subcortical functional circuits.

## Introduction

Developmental dyslexia (DD) and developmental coordination disorder (DCD) are neurodevelopmental disorders that impede the child’s ability to learn reading and to master motor skills, respectively. There is firm evidence of an overlap between these two disorders, with rates of comorbidity ranging from 30% to 50% (Chaix et al. 2007; Haslum and Miles 2007; Flapper and Schoemaker 2013a). This significant overlap has led researchers to believe in a common etiology, with shared causes to motor and speech-language abnormalities. An attractive hypothesis states that DD and DCD have impairments of the procedural learning system (Nicolson and Fawcett 2007), which subserves the learning of new, and the control of established, sensorimotor and cognitive skills, rules and habits (Ullman 2004; Knowlton et al. 2017). Impairment of this system would therefore explain deficits found in an extremely wide range of motor and perceptual skills in DCD children (Wilson et al. 2013, 2017; Adams et al. 2014), as well as secondary motor symptoms widely reported in DD (Nicholson and Fawcett 1994; Ramus et al. 2003, 2004). The procedural learning system is also proposed to subserve rule-based procedures that govern language (‘the mental grammar’), including aspects of phonology (Ullman, 2004). As such, impairments of the procedural learning system may also account for the phonological deficit, which is known to play a primary causal role in DD (Ramus 2003, 2004).

Human brain-imaging research has demonstrated that the procedural learning system in the motor domain is subtended by cortico-striatal and cortico-cerebellar networks. Cortico-striatal networks are crucial to learning and retention of sequences of new movements, while cortico-cerebellar networks are involved in motor adaptation to environmental constraints (Doyon and Benali 2005; Doyon et al. 2009; Debas et al. 2010). There also exists a dynamic reconfiguration of the activity/connectivity of these networks during the course of learning. Early to advanced visuo-motor learning involves a shift from cognitive to sensorimotor cortico-striatal circuits (Lehéricy et al. 2005; Amiez et al. 2012). In the same vein, the spatial representation of a motor sequence undergoes a reorganization from the dorsomedial (cognitive control) to the dorsolateral (sensorimotor) striatum as the sequence consolidates (Pinsard et al. 2019). Cerebellar activation during motor adaptation to a new tool spans first over posterior and anterior lobes and becomes confined to anterior lobe with time (Imamizu et al. 2000). This suggests disengagement of cognitive cerebellar territories in favor of sensorimotor ones as learning is completing. Hence, motor procedures, regardless of whether they relate to motor sequence or motor adaptation, begin as rather abstract action plans within cognitive cortico-subcortical circuits before they translate into purely motor procedures over time within sensorimotor cortico-subcortical circuits. This view is also supported by animal studies (Ashby et al. 2010). Furthermore, different types of procedural learning (e.g., motor or cognitive skills, habits) may be instantiated in different functional cortico-subcortical circuits. It is therefore important to emphasize the functional segregation of cortico-subcortical circuits as a pivotal principle underlying procedural learning.

It remains unclear whether or not impaired functional cortico-subcortical connectivity is a core hallmark of DD and/or DCD. In DD, there is direct and strong evidence of altered activity and functional connectivity within the cortical reading network, roughly a left fronto-temporo-parietal network (e.g., Shaywitz et al. 2002; Richlan et al. 2011; Richlan 2012; Finn et al., 2014; Norton et al. 2014; Schurz et al. 2015). In comparison, there is only indirect evidence of altered cortico-striatal and cortico-cerebellar network functioning. A meta-analysis found hyperactive regions in the striatum of individuals with reading disorder, and also indicated that these regions overlap mostly with a motor cortico-striatal network involved in articulatory processing (Hancock et al. 2017). Another meta-analysis reported that lobule VI and crus I of the cerebellum have decreased gray matter volume in dyslexia and that these regions mapped onto cognitive, ventral attention and frontoparietal, functional cortico-cerebellar circuits (Stoodley 2014). Hence, DD may show abnormalities in specific, motor and cognitive, cortico-subcortical functional circuits, but this is conjecture at the moment. As regards DCD, data suggest alterations in the structure and function of the cerebellum as well as a ‘disconnection syndrome’ within a large-scale cortico-subcortical functional network (e.g., Biotteau et al. 2016; Wilson et al. 2017, for reviews). However, studies are too sparse and suffer several limitations (e.g., few studies, small and inhomogeneous samples, uncontrolled comorbidities, weak neuroimaging standards; Biotteau et al. 2016) to reach more than a tentative conclusion. Finally, some authors even went one step further by formulating a neural system topography for learning disorders, in which DD and DCD are mainly related to anomalies in cortico-cerebellar and cortico-striatal circuitries, respectively (Nicolson and Fawcett 2007). However, once again, this proposition has been conjectured (i.e., deduced from behavioral symptoms) but is not based on real brain evidence.

Based on the aforementioned grounds, we built on the procedural learning deficit theory of DD and DCD and have accordingly studied cortico-subcortical functional connectivity in these disorders. We sought to determine whether different cortico-striatal and cortico-cerebellar circuits are impaired in these two disorders, and whether impairment is exacerbated when both disorders are combined (comorbidity; COM). Indeed, a number of conceptual models (Kaplan et al. 2006) and empirical studies (Jongmans et al. 2003; Ramus et al. 2003; Flapper and Schoemaker 2013b; Rao and Landa 2014) on the relationships between developmental disorders support the idea that comorbidity of disorders results in more severely affected children.

**Figure 1:**
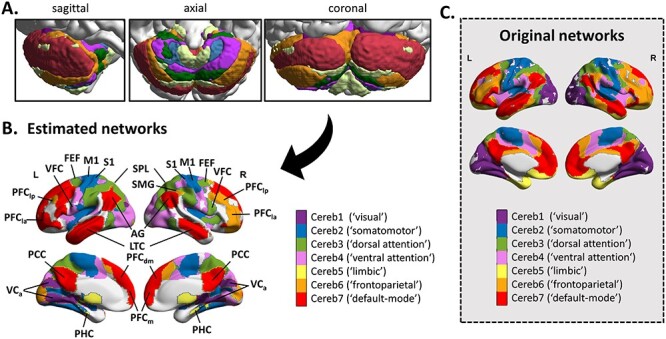
Estimated cortico-cerebellar functional circuits. (*A*) Cerebellar seeds used in the seed-to-voxels analysis. (*B*) Map of the cerebral regions functionally connected to the seeds in all children. Significant regions were obtained by applying a cluster-forming threshold *P* < 0.001 and cluster-extent threshold *P*-FDR < 0.05. (*C*) Map of the original 7 cerebral networks provided for (qualitative) comparison purposes (Buckner et al. 2011; Yeo et al. 2011). Heuristic name labels associated with the networks in the parentheses are taken from the study of Yeo et al. (2011). This should not be taken to mean that the estimated networks correspond exactly to those of Yeo et al. (2011), despite clear similarities, or that the networks code only for functions associated with their assigned name. Abbreviations: PFC_la_: lateral anterior prefrontal cortex; PFC_lp_: lateral posterior prefrontal cortex; PFC_dm_: dorsal medial prefrontal cortex; PFC_m_: medial prefrontal cortex; VFC: ventral frontal cortex; FEF: frontal eye field; M1: primary motor cortex; S1: primary somatosensory cortex; AG: angular gyrus; SMG: supramarginal gyrus; SPL: superior parietal lobule; PCC: posterior cingulate cortex; LTC: lateral temporal cortex; VC_A_: anterior visual cortex; and PHC: para-hippocampal cortex.

**Figure 2:**
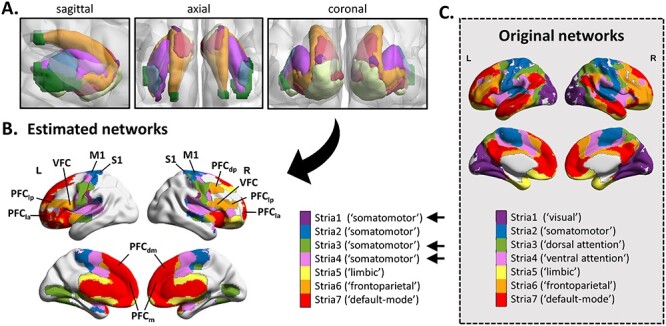
Estimated cortico-striatal functional circuits. (*A*) Striatal seeds used in the seed-to-voxels analysis. (*B*) Map of the cerebral regions functionally connected to the seeds in all children. Significant regions were obtained by applying a cluster-forming threshold *P* < 0.001 and cluster-extent threshold *P*-FDR < 0.05. (C) Map of the original 7 cerebral networks provided for (qualitative) comparison purpose (Buckner et al. 2011; Yeo et al. 2011). Heuristic name labels associated with the networks in the parentheses are taken from the study of Yeo et al. (2011). We changed the labels of some of our estimated networks due to clear topographic differences with those of the original networks (straight black arrows). Estimated networks related to stria1, stria3, and stria4 do not correspond to visual, dorsal attention, and ventral attention networks but instead are similar to the sensorimotor network. Labeling should not be taken to mean that the networks code only for functions associated with their assigned name. Abbreviations: PFC_la_: lateral anterior prefrontal cortex; PFC_lp_: lateral posterior prefrontal cortex; PFC_dm_: dorsal medial prefrontal cortex; PFC_m_: medial prefrontal cortex; VFC: ventral frontal cortex; PFC_dp_: dorsal posterior prefrontal cortex; M1: primary motor cortex; and S1: primary somatosensory cortex.

We answered these questions by examining intrinsic (rs-fMRI) functional connectivity (iFC) of multiple cortico-striatal and cortico-cerebellar networks within a large sample (*n* = 136) of 8–12-year-old typically developing children (TYP; *n* = 42) and children with DD (*n* = 45), DCD (*n* = 20), and COM (*n* = 29) scanned over two French MRI centers. iFC maps were derived from a seed-to-voxel approach that assessed correlation between the time course of striatal and cerebellar seed regions and the time course of all other voxels in the brain. We have opted for the ‘seven-network’ striatal and cerebellar seeds (Buckner et al. 2011; Choi et al. 2012), which correspond to striatal and cerebellar regions predominantly linked to the most common large-scale cerebral (visual, somatomotor, dorsal attention, ventral attention, limbic, frontoparietal control, and default-mode) resting state networks (Yeo et al. 2011). This seed-to-voxel framework enabled us to delineate the main cortico-striatal and cortico-cerebellar networks (Figs 1 and 2), especially sensorimotor and cognitive (frontoparietal) control ones, and test between-group difference for each through univariate general linear model (GLM). Further, we determined which networks discriminate the groups the most using a multivariate, sparse multiple-kernel learning (MKL; Schrouff et al. 2018), classification of the iFC maps. This method identified a subset of circuits that carried predictive information for group classification. Finally, we have undertaken a consensus analysis that uses both univariate/GLM and multivariate/MKL findings in conjunction (Varoquaux et al. 2018), with the aim of reaching the most reliable conclusion as regards specific cortico-striatal and cortico-cerebellar functional circuits that are affected in DD and/or DCD.

## Materials and Methods

### Participants

About 136 right-handed children between the ages of 8 and 12 years participated in this study, including 42 TYP children (10.10 years ± 1.16 year, 20 girls), 45 DD children (10.22 years ± 1.09 year, 20 girls), 20 DCD children (9.96 years ±1.23 year, 6 girls), and 29 COM children (10.19 years ± 1.31 year, 10 girls). About half of the children were recruited in Toulouse area and the other half in Aix-Marseille area (Toulouse/Aix-Marseille; TYP: 21/21; DD: 25/20; DCD: 11/9; COM: 15/14).

All children underwent neuropsychological assessment, including tests of intellectual abilities (WISC-IV; Wechsler, 2005), reading skills (Alouette test, Lefavrais 2005; ODEDYS-1 battery, Jacquier-Roux et al. 2005), and motor skills (Movement Assessment Battery for Children, M-ABC French translation, Soppelsa and Albaret 2004). DCD children all met the DSM-V diagnostic criteria for discrete motor disorder: (1) M-ABC-1 was below the fifth percentile; (2) treated for a motor coordination problem by a pediatric physical therapist due to a persistent interference with activities of daily living; (3) no sign of intellectual disability (IQ score > 70 or subtest similarities and picture concepts scaled scores > 7 in WISC-IV); and (4) no visual impairments or neurological conditions that could affect their motor abilities. Children with DD met the DSM-V diagnostic criteria for specific learning disorder with impairment in reading: (1) their accuracy and speed when reading were significantly low, that is a performance for reading isolated irregular words or logatoms (ODEDYS-1) 1.5 standard deviation (SD) below the mean and an Alouette text reading speed score or accuracy score 1 SD below the mean; (2) they were treated for a reading problem by a pediatric speech therapist; and (3) they had no diagnosis of any significant medical or social condition known to affect reading abilities and they did not have any sign of intellectual disability (WISC total IQ > 70 or/and subtest similarities and picture concepts scaled scores > 7). To avoid any overlap between children with DD and DCD, additional inclusion criteria included an M-ABC total percentile score > 20th percentile for DD children and reading (accuracy and speed) scores above 0.5 SD below the mean on both the Alouette and ODEDYS-1 (irregular words or logatoms) tests for DCD children. These criteria also applied for TD children, who were also screened on intellectual abilities (IQ score > 70 or subtest similarities and picture concepts scaled scores > 7 in WISC-IV). COM children combined DSM-V diagnostic criteria for both discrete motor disorder and learning disorder with impairment in reading mentioned above. DD children received multicomponent interventions targeting fluency, reading comprehension, phonological skills, and vocabulary study. Treatments for DCD children involved process-oriented, task-oriented, and conventional physical and occupational therapies. Children with suspicion of attention deficit hyperactivity disorder (ADHD) were excluded from the study as assessed through parent and clinician ratings on the DSM-V diagnostic criteria for ADHD (< six inattention and six hyperactive/impulsive symptoms). This exclusion criterion was particularly important to consider given the aggravating impact of ADHD on DD and DCD (Crawford et al. 2006; Chaix et al. 2007).

The study was approved by local ethics committees (CPP) in Toulouse and Aix-Marseille and was conducted in accordance with the Declaration of Helsinki. We obtained written informed consent from the parents and their children.

### MRI Acquisition

*Toulouse:* MRI images were acquired at the Toulouse NeuroImaging Center using a Philips Achieva dStream 3.0 T MRI scanner equipped with a 32-channel head coil. Rs-fMRI scans were collected using an echo planar imaging (EPI) sequence: Time repetition (TR)/time echo (TE) = 3000/40 ms, flip angle (FA) = 90°, field of view (FOV) = 240 mm, matrix = 80 × 80, voxel size = 3.0 × 3.0 × 3.0 mm, 46 axial slices. The scan session was 600 s long and included 200 volumes. Participants received instructions to keep their eyes closed, to think nothing in particular, and to not move. T1-weighted images were acquired using a fast field echo sequence: TR/TE = 8.1/3.7 ms, FOV = 240 mm, matrix = 240 × 240 × 170, voxel size = 1 × 1 × 1 mm, and 170 sagittal slices.

*Aix-Marseille*: MRI images were acquired at the radiology department of the University Medical Centre La Timone using a Siemens Magnetom Skyra 3.0 T MRI scanner equipped with a 32-channel head coil. Rs-fMRI scans were collected using an EPI sequence: TR/TE = 2540/30 ms, FA = 90°, FOV = 192 mm, matrix = 64 × 64, voxel size = 3.0 × 3.0 × 3.0 mm, and 45 axial slices. A scan session was 635 s long and included 250 volumes. Participants received similar instructions as in Toulouse for rs-fMRI. T1-weighted images were acquired using a magnetization-prepared rapid gradient echo (MPRAGE) sequence: TR/TE = 1900/2.5 ms, inversion time = 993 ms, FA = 9°, FOV = 230 mm, matrix = 256 × 256, voxel size = 0.9 × 0.9 × 0.9 mm, and 192 sagittal slices.

### Image Preprocessing

Rs-fMRI images were preprocessed using functions of SPM12 (Wellcome Trust Centre for Neuroimaging; www.fil.ion.ucl.ac.uk/spm) including the following steps: 1/realignment and unwarp, 2/slice-timing correction, 3/direct normalization to functional MNI space, 4/detection of functional outliers (ART-based censoring; www.nitrc.org/projects/artifact_detect), and 5/smoothing with an 8-mm kernel. Direct normalization (via segmentation) to the T1 MNI template image was also applied to the structural image as part of preprocessing. In step 4, an image was identified as an outlier if the framewise displacement (FD) of the head position was greater than 0.5 mm from the previous frame, or if the global mean intensity in the image was greater than 3 SDs from the mean image intensity for the entire resting session. These conservative threshold values have been used in previous developmental studies (i.e., children cohorts)—whose preprocessing/denoising procedures were very similar to ours—to attenuate motion-related artifacts (e.g., Chai et al. 2014, 2016; Cignetti et al. 2018a; Whitfield-Gabrieli et al. 2019). This censoring strategy identified on average 18% of invalid volumes in our entire sample and yielded a reduction in FD of the head at a value close to 0.2 mm in any groups (Table 1). These values roughly correspond to generally accepted norms (e.g., Power et al. 2012, 2014).

**Table 1:** Summary statistics of motion censoring

	% Invalid scans	FD before censoring (mm)			FD after censoring (mm)		
		ART	Power	Jenkinson	ART	Power	Jenkinson
TYP	16 (24)	0.41 (0.59)	0.45 (0.54)	0.24 (0.31)	0.15 (0.07)	0.19 (0.09)	0.09 (0.04)
DD	14 (16)	0.28 (0.21)	0.32 (0.23)	0.17 (0.13)	0.14 (0.04)	0.17 (0.04)	0.08 (0.02)
DCD	26 (23)	0.60 (0.67)	0.65 (0.68)	0.34 (0.35)	0.17 (0.04)	0.21 (0.06)	0.11 (0.03)
COM	18 (18)	0.30 (0.25)	0.34 (0.28)	0.18 (0.15)	0.15 (0.04)	0.18 (0.06)	0.09 (0.05)

ART’s FD measure has been used to censor invalid scans. Power (Power et al. 2012) and Jenkinson (Jenkinson et al. 2002) FDs are also provided for comparison purposes. Values are mean (SD).

### Denoising

BOLD time series were denoised using the aCompCor method (Behzadi et al. 2007; Chai et al. 2012, 2014) of the CONN toolbox (www.nitrc.org/projects/conn;Whitfield-Gabrieli and Nieto-Castanon 2012). This consisted in regressing out from the BOLD time series at each voxel temporal confounds, including five principal components of the signals from white matter and cerebrospinal masks with partial volume correction applied. Head motion parameters (three translation and three rotation parameters, plus another six parameters representing their first-order temporal derivatives), and outlier images were also regressed out from the BOLD time series. The BOLD time series were finally band-pass filtered (0.008–0.09 Hz).

### Functional Connectivity

Functional connectivity was calculated as the Pearson’s correlation coefficient between denoised average BOLD time series computed across all the voxels within each seven-network cerebellar (Buckner et al. 2011) and striatal (Choi et al. 2012) seeds and every other voxel in the brain (seed-to-voxel analysis). These seeds have been previously delineated using a winner-takes-all algorithm that associated each voxel of the cerebellum and of the striatum with a cortical network (defined in Yeo et al. 2011) with the most similar profile of connectivity. Accordingly, names of the seeds have been originally assigned based on the cortical networks best corresponding to them, namely visual, somatomotor, dorsal attention, ventral attention, limbic, frontoparietal, and default-mode. However, some of our estimated cortico-cerebellar and cortico-striatal networks did not correspond to these reference labels, which led us to change the nomenclature. This is explained in the results, section ‘Cortico-cerebellar and cortico-striatal estimated functional networks’ and illustrated in Figures 1, 2, S1, and S2. Finally, correlation coefficients were converted to normally distributed *z*-scores using the Fisher transformation.

### GLM Analysis

*T*-tests and *F*-tests were run on the first-level correlation maps. One-sample *T*-tests produced group level correlation maps for each seed in the entire sample (i.e., all groups combined), with the aim of delineating the cortico-striatal and cortico-cerebellar functional circuits and comparing them (qualitatively) with previous results from the literature (Buckner et al. 2011). ANCOVA *F*-tests examined between-group differences for each seed-based correlation map, including age, gender, and GCOR (average global correlation) as covariates. Correction for GCOR at the group level is a conservative approach to reduce global variations in BOLD signal due to noise and nuisance sources (Saad et al. 2013), like the factor center in our experiment. Note that we did not judged necessary to include motion-related measures in group-level analysis because neither the percentage (arcsin transformed) of invalid scans (*F*_(3,135)_ = 1.58; *P* = 0.19) nor the mean FD after censoring (*F*_(3,135)_ = 1.63; *P* = 0.18) significantly differed between groups. Furthermore, we tested through bivariate regression any association between functional connectivity of the cortico-striatal and cortico-cerebellar functional maps and behavioral scores that capture motor and reading skills. These scores were the M-ABC (expressed as percentile) and the first principal component (99% of the total variance explained) of a set of reading scores (i.e., Alouette text reading speed and accuracy and reading isolated irregular words and logatoms in ODEDYS-1). All analyses were thresholded by applying cluster-forming threshold *P* < 0.001 and cluster-extent threshold *P*-FDR < 0.05. FDR was also adjusted on the entire set of seeds (*n* = 14) using the Benjamini and Hochberg procedure. Both adjusted and nonadjusted *P*-values are reported in the manuscript.

### MKL Analysis

We performed MKL analysis using PRoNTo toolbox (http://www.mlnl.cs.ucl.ac.uk/pronto;Schrouff et al. 2013, 2018). This machine learning approach is based on the formulation of a support vector machine (SVM) classifier, but rather than relying on a single kernel to separate the data into classes, it forms an optimal kernel from a linear combination of kernels (Rakotomamonjy et al. 2008; Gönen and Alpaydin 2011; Schrouff et al. 2018), here corresponding to our connectivity maps. To determine whether and which correlation maps distinguished between the four groups of children, we implemented six binary MKL models (i.e., TYP vs DD, TYP vs DCD, TYP vs COM, DD vs DCD, DD vs COM, and DCD vs COM), thus transforming the four-class problem into six binary learning problems (i.e., class binarization). In all MKL models, the first step consisted of building as many kernel matrices of size *n* x *n* (*n* being the number of subjects) as seed-based correlation maps (i.e., 14), which represented the similarity between subjects for each map. To this end, signal in voxels of each correlation map was extracted and concatenated in a feature vector of size *d* x 1, with *d* the number of voxels, for each individual. Each vector was then associated to a label (here a categorical value, given the above binary classification). Finally, a linear kernel (i.e., dot product) was then built from the feature vectors for each correlation map. Kernels were mean-centered and normalized before entering the MKL model. The built kernels and their associated labels were then submitted to an SVM classifier that defined a decision boundary separating the labels per kernel (Schrouff et al. 2018). More specifically, this classifier estimated/optimized model parameters (*w_m_*) to determine decision boundaries for classification, one per kernel *m*, and weighted them (by a parameter *d_m_*) to provide a final decision function. Regularization constraints in the considered classifier enforced sparsity on the kernels, so that only some kernels had non-null contribution *d_m_* to the final decision function. Details on regularization and optimisation of MKL can be found elsewhere (Rakotomamonjy et al. 2008, Schrouff et al. 2018). In sum, the classifier identified which kernels (here connectivity maps) contributed the most to the classification. In addition, the models weights *w_m_* were mapped onto brain volume to display the decision function at the voxel level (i.e., weight maps). Model performance was assessed through a leave-one-subject-per-class-out crossvalidation scheme, which consisted in leaving out a single subject from each class at a time for testing and training with the remaining subjects. This crossvalidation scheme was the most appropriate given that we have matched subjects across groups based on MRI center, gender, and age, to avoid unbalanced datasets and biasing our prediction models towards the more common classes. Thus, subject matching involved under-sampling the largest groups, including 42 samples for the TYP vs DD model, 20 samples for the TYP vs DCD model, 29 samples for the TYP vs COM model, 20 samples for the DD vs DCD model, 29 samples for the DD vs COM model, and 20 samples for the DCD vs COM model (see Table 2). Balanced accuracy served as the model performance metric, computed as the mean of the class accuracies (corresponding to the sensitivity and specificity). A *P*-value associated with balanced accuracy was estimated using 1000 permutations of the training labels, with any value smaller than 0.05 reported as being significant. However, given that we implemented several MKL models, we also controlled FDR using the Benjamini and Hochberg procedure, and reported adjusted *P*-value when primary *P*-value was below 0.05.

**Table 2:** Model performance for the MKL model distinguishing between the different classes of children

Model MKL	Balanced accuracy (%)	True positives/Total positives	True negatives/Total negatives	AUC_ROC_
[TYP] vs. [DD]	44.05 (*P* = 0.75)	18/42	19/42	0.41
[TYP] vs. [DCD]	57.50 (*P* = 0.27)	14/20	9/20	0.60
[TYP] vs. [COM]	75.86 (*P* = 0.005)	23/29	21/29	0.80
[DD] vs. [DCD]	45.50 (*P* = 0.76)	9/20	8/20	0.51
[DD] vs. [COM]	46.55 (*P* = 0.67)	13/29	14/29	0.52
[DCD] vs. [COM]	47.50 (*P* = 0.61)	11/20	8/20	0.41
[TYP] vs. [DCD-COM]	71.43 (*P* = 0.001)	30/42	30/42	0.75
[TYP] vs. [DD-COM]	63.10 (*P* = 0.04)	28/42	25/42	0.67

All significant MKL models survived FDR correction. Binary classifier performance is summarized through 3 measures: balanced accuracy, true positives/negatives that represent the number of children classified correctly as belonging to class1/class2, and the AUC of the receiver operator characteristic curve (1 represents perfect performance, 0.5 represents random performance).

### GLM–MKL Consensus

Multivariate approaches, such as MKLs, are thought to be more specific (more localized outcomes) and sensitive (easier detection of significant outcomes) than univariate approaches, such as the GLMs. But there is also an interpretability issue with multivariate approaches due to the contribution of noise in the decoding process of linear classifiers (Haufe et al. 2014). Therefore, several authors recommend using both approaches in conjunction to come up with more robust and interpretable conclusions (Schrouff et al. 2016, 2018; Varoquaux et al. 2018). However, performing conjunction analysis between data that use different statistics (in our case F-values and SVM weights) is not straightforward and requires some rescaling to bring those statistics into a comparable scale. In our case, the conjunction (or consensus) focused on weight maps of MKL models having performed significantly above chance level and F-maps of GLM having demonstrated significant between-group differences. First, we computed *z*-scores for the weight maps by dividing the classifier weights by their standard error (obtained by crossvalidation). Then, we normalized *z*-scores of the weight maps and *F*-scores of the *F*-maps by rescaling them on the same [0–1] range, using the formula (*x* – *x*_min_)/(*x*_max_ – *x*_min_). Finally, we thresholded the maps using a 95% percentile threshold and intersected them to identify common voxels.

## Results

### Cortico-Cerebellar and Cortico-Striatal Estimated Functional Networks

Cerebellar seeds showed segregated functional connectivity pathways with cortical regions (Fig. 1), including connections to association visual cortices (visual network; purple), connections to primary motor and somatosensory cortices (somatomotor network; blue), connections to superior parietal lobules and frontal eye fields (dorsal attention network; green), connections to temporo-parietal junction and ventral frontal regions (ventral attention network; violet), connections to para-hippocampal cortices (limbic network; cream), connections to lateral prefrontal cortices and inferior parietal lobules (frontoparietal network; orange), and connections to ventromedial prefrontal and posterior cingulate cortices and angular gyri (default-mode network; red). Overall, the topography of the above cortico-cerebellar networks matched well the original seven network topography (Buckner et al. 2011; Yeo et al. 2011), although there were also some regional differences. First, occipital regions figured surprisingly in all our estimated functional networks. Second, the cortico-cerebellar ventral attention network was not limited to temporo-parietal and ventral frontal regions but also included motor and somatosensory cortical regions (Fig. S1). As such, the ventral attention network does not correspond exactly to his assigned name but embeds also a clear sensorimotor dimension. This underlines that the reference labels associated with the networks are only heuristic labels and should not be taken to mean that the networks code solely for these functions.

The topography of our cortico-striatal networks was different from that of the original seven networks (Fig. 2). Only the cortico-striatal somatomotor network (blue) corresponded well to that in the literature, spanning primary motor, and somatosensory cortical regions. We also identified cortico-striatal frontoparietal (orange) and default-mode (red) networks, yet incompletely. The frontoparietal network spanned both lateral prefontal cortices, but not posterior parietal cortices. The default mode network involved ventromedial prefrontal regions but lacked classical posterior parietal and posterior cingulate regions. The limbic network (cream) was almost inexistent, with only a few voxels spanning the parahippocampal gyrus. Finally, striatal seeds assigned to vision (purple), ventral attention (violet), and dorsal attention (green) all connected to motor and somatosensory regions, hence revealing overlapping sensorimotor cortico-striatal connectivity patterns (Fig. S2). Thus, the topographic distribution of the cortico-striatal functional connectivity was expressed in a few separate networks, mainly sensorimotor, frontoparietal, and default-mode networks.

### Impact of DD and DCD on Cortico-Cerebellar and Cortico-Striatal Functional Circuits

**Figure 3:**
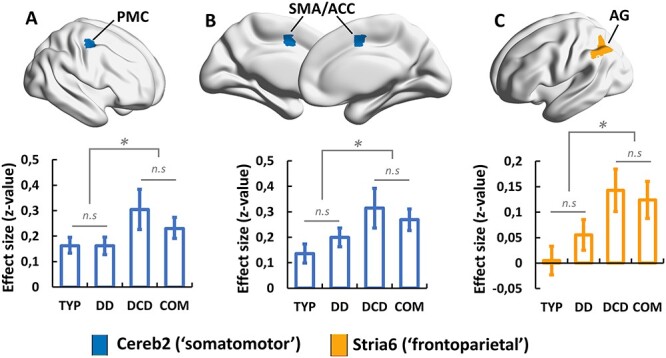
Cerebral regions of the cortico-subcortical functional circuits that expressed any difference between TYP, DD, DCD, and COM children. All results were obtained using a cluster-forming threshold *P* < 0.001 and a cluster-extent threshold *P*-FDR < 0.05 for whole brain, and survived FDR correction on the entire set of networks. Effect sizes are provided as mean ± 90% CI. ^*^: group means are significantly different. PMC: premotor cortex; SMA: supplementary motor area; ACC: anterior cingulate cortex; and AG: angular gyrus.

We found main effects of group in somatomotor cortico-cerebellar and frontoparietal cortico-striatal circuits (Fig. 3). With respect to the somatomotor cortico-cerebellar connectivity, there was a significant cluster (*P*-FDR = 0.0035; adjusted *P*-FDR = 0.025) in the right dorsal premotor cortex (MNI *X*, *Y*, *Z* coordinates: 36, −10, 54) and another significant cluster (*P*-FDR = 0.0038; adjusted *P*-FDR = 0.026) at the boundary between the supplementary motor area and the dorsal anterior cingulate cortex (MNI *X*, *Y*, *Z* coordinates: 6, −2, 46). For both, posthoc pairwise *t*-tests revealed a stronger iFC (hyperconnectivity) in DCD and COM children compared with TYP and DD children. Regarding the frontoparietal cortico-striatal connectivity, there was a significant cluster (*P*-FDR = 0.000391; adjusted *P*-FDR = 0.0055) in the left angular gyrus (MNI *X*, *Y*, *Z* coordinates: −46, −56, 42), where iFC was also stronger (hyperconnectivity) in DCD and COM children compared with TYP and DD children. Note that connectivity about this posterior parietal region was close to zero-level in both TYP and DD children, which explains the fact that the region was not previously identified as being part of the frontoparietal cortico-striatal circuit at the entire sample level. In sum, somatomotor cortico-cerebellar and frontoparietal cortico-striatal network hyperconnectivity sets apart conditions with DCD from DD and TYP conditions. Besides, there was no additive effect of comorbidity on hyperconnectivity.

**Figure 4:**
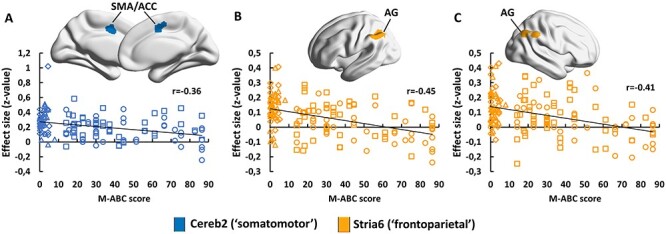
Cerebral regions of the cortico-subcortical functional circuits that expressed a significant correlation with M-ABC score. All results were obtained using a cluster-forming threshold *P* < 0.001 and a cluster-extent threshold *P*-FDR < 0.05 for whole brain. Only correlations between M-ABC and frontoparietal cortico-striatal connections survived FDR correction on the entire set of networks. Circle: TYP; Square: DD; Diamond: DCD; Triangle: COM. SMA: supplementary motor area; ACC: anterior cingulate cortex; and AG: angular gyrus.

Interestingly, we further found that two of the functional connectivity pathways that expressed a group effect, namely the one established between the cerebellum and the border region between the supplementary motor area and dorsal anterior cingulate cortex and the one linking the striatum and the left angular gyrus, also showed significant correlations (*P*-FDR = 0.028; adjusted *P*-FDR = 0.16 and *P*-FDR = 0.000028; adjusted *P*-FDR = 0.0004, respectively) with the M-ABC score (Fig. 4). For both relationships, the higher the iFC the poorer the children motor control (i.e., the lower the M-ABC). Hence, somatomotor cortico-cerebellar and frontoparietal cortico-striatal network connectivity indexes motor control capabilities. We further found a significant negative correlation between functional connections established between the cerebellum and the right angular gyrus (MNI *X*, *Y*, *Z* coordinates: 50, −55, 52) and the M-ABC (*P*-FDR = 0.000087; adjusted *P*-FDR = 0.0012), indicating that not only the left angular gyrus but also the right angular gyrus is associated with motor control. Altogether, the above findings paint the consistent picture that some somatomotor cortico-cerebellar and frontoparietal cortico-striatal functional connections are central to motor control, which once altered (here, overconnected) are hallmarks of DCD disorder. Note that correlation analysis between connectivity in these regions and the reading score did not reveal any significant result.

### Decoding Children Populations from Cortico-Cerebellar and Cortico-Striatal Functional Connectivity

Among all models, only the one classifying TYP and COM children was found to be significant (*P* = 0.005) with accuracy and area under curve (AUC) values of 75.86% and 0.8, respectively (Table 2). This result remained significant (adjusted *P*-FDR = 0.03) when correcting *P*-value using the Benjamini and Hochberg procedure to address multiple testing across MKL models. There were four kernels that contributed the most to the decision function of the TYP-COM MKL model, including three cortico-cerebellar iFC maps—default-mode (~44%), dorsal attention (~13%), and ventral attention (~11%)—and the frontoparietal cortico-striatal (~12%) iFC map (Table 3). At first sight, it may seem surprising not to find somatomotor cortico-cerebellar iFC map among the kernels that contributed the most to the TYP-COM MKL model while GLM identified difference between TYP and COM children in this functional pathway. However, it should be recalled that ventral attention cortico-cerebellar network aggregated both ventral attention and somatomotor regions (see also weight maps results below). Accordingly, the true conclusion from MKL should be that several cortico-cerebellar functional circuits believed to be associated with default-mode, somatomotor, dorsal attention, and ventral attention functions and a cortico-striatal circuit likely to be associated with frontoparietal control mostly contribute to differentiate TYP and COM children. As such, it is interesting to note that MKL revealed a much more complex picture of cortico-subcortical connectivity differences between TYP and COM than the one derived from the GLM, suggesting a complementarity between the two methods that we further capitalize on below (see Consensus between GLM and MKL approaches).

**Figure 5:**
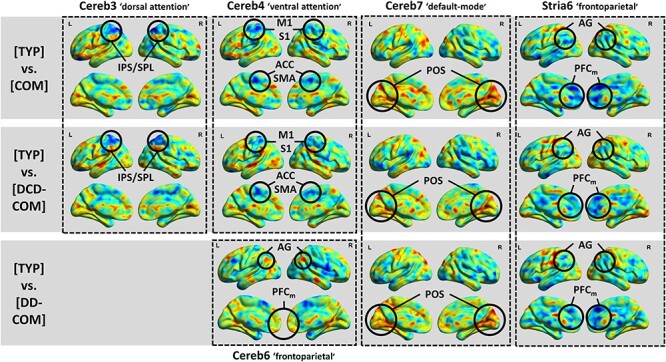
Weight images at the voxel level for the TYP-COM, TYP-[DCD-COM], and TYP-[DD-COM] MKL models. Only images for kernels (cortico-subcortical circuits) with the greatest contribution (>10%) to the models are presented. Voxels increasing the signed distance from the classification boundary the most, either toward TYP (voxels in warm colors) or COM, DCD-COM, and DD-COM (voxels in cold colors), have been circled. Voxels in green have null contribution to the model. Abbreviations: PFC_m_: medial prefrontal cortex; M1: primary motor cortex; S1: primary somatosensory cortex; SMA: supplementary motor area; ACC: anterior cingulate cortex; AG: angular gyrus; IPS: intraparietal sulcus; SPL: superior parietal lobule; and POS: parieto-occipital sulcus.

**Table 3:** Kernel contribution (*d_m_*) to the MKL models performing significantly above chance level, along with the expected ranking, across folds

Model MKL [TYP] vs. [COM]			Model MKL [TYP] vs. [DCD-COM]			Model MKL [TYP] vs. [DD-COM]		
ROI#	*d_m_* (%)	ER	ROI#	*d_m_* (%)	ER	ROI#	*d_m_* (%)	ER
Cereb7 (‘default-mode’)	44.4370	14	Cereb7 (‘default-mode’)	39.8886	14	Cereb7 (‘default-mode’)	46.3638	14
Cereb3 (‘dorsal attention’)	13.4819	12.4103	Cereb4 (‘ventral attention’)	13.1845	12.2619	Cereb6 (‘frontoparietal’)	14.7626	12.5476
Stria6 (‘frontoparietal’)	12.4501	11.7586	Stria6 (‘frontoparietal’)	12.2180	11.9048	Stria6 (‘frontoparietal’)	13.8192	12.3571
Cereb4 (‘ventral attention’)	11.0945	11.1379	Cereb3 (‘dorsal attention’)	10.4024	11.0952	Cereb3 (‘dorsal attention’)	7.0694	10.2857
Stria7 (‘default-mode’)	8.1143	9.8966	Cereb2 (‘somatomotor’)	7.7157	9.6667	Cereb4 (‘ventral attention’)	7.0532	9.9762
Stria4 (‘somatomotor’)	6.1296	8.7931	Cereb6 (‘frontoparietal’)	6.0691	8.9524	Cereb2 (‘somatomotor’)	4.0890	8.0714
Cereb1 (‘visual’)	3.9389	7.6552	Stria4 (‘somatomotor’)	5.9496	8.4048	Cereb5 (‘limbic’)	3.2966	7.6667
Cereb6 (‘frontoparietal’)	0.3346	1.4828	Stria7 (‘default-mode’)	3.4399	6.7619	Stria7 (‘default-mode’)	3.1173	7.2381
Cereb5 (‘limbic’)	0.0191	0.2414	Cereb5 (‘limbic’)	0.4929	2.3810	Stria5 (‘limbic’)	0.3052	2.0476
Cereb2(‘frontoparietal’)	0	0	Cereb1 (‘visual’)	0.3341	2.3571	Stria4 (‘somatomotor’)	0.1237	1.0714
Stria1 (‘somatomotor’)	0	0	Stria5 (‘limbic’)	0.3052	2.0476	Cereb1 (‘visual’)	0	0
Stria2 (‘somatomotor’)	0	0	Stria1 (‘somatomotor’)	0	0	Stria1 (‘somatomotor’)	0	0
Stria3 (‘somatomotor’)	0	0	Stria2 (‘somatomotor’)	0	0	Stria2 (‘somatomotor’)	0	0
Stria5 (‘limbic’)	0	0	Stria3 (‘somatomotor’)	0	0	Stria3 (‘somatomotor’)	0	0

Expected ranking was computed as the average of the ranking across folds, thus evaluating the stability of the kernel contribution. Only the kernels that carry the most predictive information about group classification (>10% of contribution to MKL) are discussed in the text.

We also built extra MKL binary models distinguishing TYP children and either DD-COM children or DCD-COM children (Table 2). The idea behind mixing COM children with either DD or DCD children was to tease out which of the DD or DCD disorder was the most contributing to the differentiation between TYP and COM children reported above (i.e., posthoc testing within the MKL framework). Mixtures of DD-COM and DCD-COM children included the same number of DD or DCD children (*n* = 16) grouped with the COM children (*n* = 26). DD-COM and DCD-COM children were matched with TYP children based on MRI center, gender and age. Both models were significant (*P* and adjusted *P*-FDR = 0.04 for TYP-[DD-COM] model and *P* = 0.001 and adjusted *P*-FDR = 0.002 for TYP-[DCD-COM] model). The differences were in prediction accuracy and contributing kernels to the models. Accuracy and AUC values for the classification of TYP and DCD-COM children remained very close to those of the TYP-COM model (71.43% and 0.75 versus 75.86% and 0.8, respectively; Table 2). Likewise, kernels with the most important contribution to the TYP-[DCD-COM] model were the same as those of the TYP-COM model, including the default-mode (~40%), dorsal attention (~10%), and ventral attention (~13%) cortico-cerebellar iFC maps as well as the frontoparietal cortico-striatal (~12%) iFC map (Table 3). Therefore, the same conclusion is reached, saying that cortico-cerebellar functional circuits believed to be associated with multiple functions (default-mode, dorsal attention/somatomotor, ventral attention) and a cortico-striatal circuit likely associated with frontoparietal control mostly differentiate TYP and DCD-COM children. On the other hand, accuracy and AUC values for the TYP-[DD-COM] model decreased compared with those of the TYP-COM model (63.10% and 0.67 versus 75.86% and 0.8, respectively; Table 2). There were also some changes in the kernels that contributed the most to the MKL model, in that only default-mode cortico-cerebellar iFC map (~46%) and frontoparietal cortico-striatal iFC map (~14%) still conveyed most of the predictive information, altogether with the frontoparietal cortico-cerebellar iFC map (~15%). Both dorsal and ventral attention cortico-cerebellar iFC maps still conveyed some predictive information, but to a lower extent (~7%). In sum, the distinction between TYP and COM appears to be driven more by the DCD condition than by the DD condition. As such, DCD rather than DD may be characterized by impaired connectivity in multiple cortico-cerebellar (default-mode, dorsal attention/somatomotor, ventral attention) and cortico-striatal (fronto-parietal) networks.

Weight maps representing the relative contribution of voxels to all predictive models (i.e., TYP-COM, TYP-[DCD-COM], and TYP-[DD-COM]) attributed high negative weights to voxels in right and left posterior parietal and medial prefrontal cortices for the cortico-striatal connectivity (Fig. 5; frontoparietal map) and high positive weights to voxels close to the parieto-occipital junction as regards the cortico-cerebellar connectivity (Fig. 5, default-mode map). This means that increase in signal at these regions increases the distance from the classification boundary the most, moving towards either COM, DCD-COM or DD-COM children for the fronto-parietal regions and moving towards TYP children for the parieto-occipital junction. In addition, weight maps of TYP-COM and TYP-[DCD-COM] models attributed high negative weights to voxels in medial (supplementary motor area/anterior cingulate cortex) and lateral (premotor and primary motor and somatosensory cortices) central regions (Fig. 5, ventral attention map) and in the superior parietal lobule (Fig. 5, dorsal attention map), with any increase in signal at these regions increasing the probability of being class COM and DCD-COM.

### Consensus Between GLM and MKL Approaches

**Figure 6:**
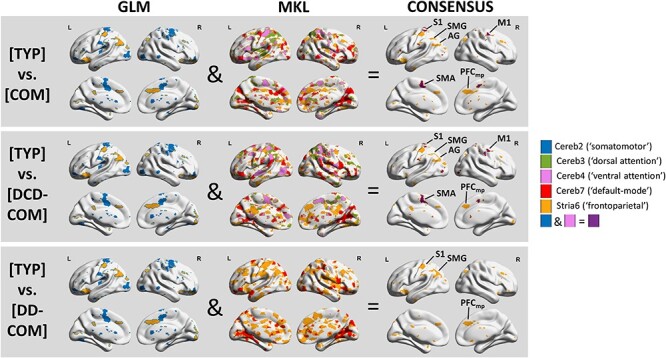
Maps for consensus between GLM and MKL. These maps single out a set of cerebral regions associated to cortico-subcortical circuits that distinguishes between groups of children. Abbreviations: S1: primary somatosensory cortex; M1: primary motor cortex; SMA: supplementary motor area; AG: angular gyrus; SMG: supramarginal gyrus; and PFCmp: medial posterior prefrontal cortex.

As a final step, we captured the consensus between the GLM and MKL approaches. Results showed that COM children differ from TYP children mainly at (i) sensorimotor cortico-cerebellar connections projecting onto the left primary somatosensory cortex, the right motor cortex and the right and left supplementary motor and anterior cingulate areas, and (ii) frontoparietal cortico-striatal connections projecting onto the left angular and supramarginal gyri and the right and left medial prefrontal cortices (Fig. 6). Results were exactly the same for the differentiation between TYP and DCD-COM children while only the frontoparietal cortico-striatal connections still differentiated TYP and DD-COM children. This leads to the conclusion that impaired sensorimotor cortico-cerebellar connections and frontoparietal cortico-striatal connections is characteristic of DCD, and that impairments in frontoparietal cortico-striatal connections may also be shared by DD.

## Discussion

Our study investigated whether intrinsic cortico-subcortical functional circuits are impaired in DD and/or DCD and whether some impairments in these circuits are more related to either of the disorders. The goal was to address the assumption according to which any neurodevelopmental disorder, including DD and DCD, would have impairments in cortico-subcortical circuits (Nicolson and Fawcett 2007). Our findings demonstrated for the first time that intrinsic cortico-subcortical functional connectivity is affected in children with DCD and DD-DCD (i.e., COM), including abnormalities in cortico-cerebellar connections targeting sensorimotor regions (S1, M1, SMA/ACC) and cortico-striatal connections mapping onto posterior parietal cortex (AG, SMG). On the other hand, we did not find clear evidence of a functional connectivity deficit in DD, although our data cannot totally exclude the possibility that frontoparietal cortico-striatal functional network is marginally affected in DD. As such, our study supports the proposal of impaired cortico-subcortical functional circuits as a core feature of DCD phenotype, but this may not apply as a general rule for any neurodevelopmental disorder. For DCD, this finding complements previous studies that foreshadowed atypical functional brain networks involving cortical (e.g., parietal) and subcortical (cerebellum, basal ganglia) regions (Biotteau et al. 2016; Wilson et al. 2017; Fuelscher et al. 2018). In contrast, the null outcome is startling for DD given that previous studies reported impaired regions in the striatum and the cerebellum, which also overlapped with known cortico-striatal (motor) and cortico-cerebellar (ventral attention, frontoparietal control, default-mode) functional pathways (Stoodley 2014; Hancock et al. 2017). However, this was not direct evidence of impaired cortico-subcortical functional connectivity. Besides, these previous studies considered DD children on the single basis of their performance in reading while we also controlled for motor deficits. This is critical given that 30–50% of DD children also show difficulties in fine and/or gross motor coordination (Chaix et al. 2007; Haslum and Miles 2007; Flapper and Schoemaker 2013a). It is therefore possible that they included a significant proportion of comorbid, DD-DCD, children who may have biased the results.

Functional connectivity impairments in sensorimotor cortico-cerebellar and frontoparietal cortico-striatal networks in the presence of DCD altogether with the relationship found between functional connectivity of these pathways and M-ABC offer an explanatory framework to the disease clinical picture. Pathways linking the cerebellum, the basal ganglia and precentral /postcentral cortical regions are common pathways of goal-directed motor actions (Scott 2004). As such, performance difficulties exhibited by DCD children in a wide range of movements, in particular eyes movements, reaching, grasping, bimanual lifting, manual interception, posture, and gait (Wilson et al. 2013, 2017), could stem from the reported connectivity impairments. Relatedly, a growing literature reports atypical predictive motor control in DCD, which impacts negatively both motor planning and online control of movements (Jover et al. 2010; Adams et al. 2014; Wilson et al. 2013, 2017; Cignetti et al. 2018b). Internal models subtending predictive motor control have been linked to the cerebellum (Wolpert et al. 1998; Blakemore et al. 1999, 2001; Imamizu et al. 2000), the somatosensory cortex (Shergill et al. 2013), and the posterior parietal cortex (Wolpert et al. 1998; Desmurget and Sirigu 2009). Accordingly, abnormalities in functional cerebellar and striatal connections projecting onto sensorimotor and posterior parietal regions, respectively, are compatible with the internal modeling hypothesis in DCD. Besides, DCD children have difficulties in learning new motor skills, and both skill acquisition and retention processes seem to be affected (King et al. 2011; Biotteau et al. 2015). In recent years, brain studies have shown dynamic reconfiguration of the activity/connectivity of cortical-subcortical networks during the course of motor learning, from cognitive control to sensorimotor networks as learning moves from acquisition to retention (Lehéricy et al. 2005; Amiez et al. 2012; Pinsard et al. 2019). As such, difficulties in acquisition and retention of motor skills in DCD may be engendered by impaired cortico-subcortical functional connections projecting onto sensorimotor and posterior parietal regions as reported in the present study.

Interestingly, a previous study on individuals with autism spectrum disorder (ASD) reported resting state overconnectivity in cortico-subcortical (both cerebellum and basal ganglia) pathways targeting sensorimotor regions (Cerliani et al. 2015). The authors related this finding to the atypical sensorimotor behavior commonly reported in ASD. Hence, cerebellar and striatal overconnections with the sensorimotor (motor/somatosensory) cortical regions may be the hallmark of neurodevelopmental disorders characterized by sensorimotor dysfunction, either DCD or ASD. The authors further proposed that overconnectivity might be due to disrupted GABAergic functioning. Cortico-cerebellar overconnectivity could stem from a disinhibition of the deep cerebellar nuclei due to the loss of GABAergic Purkinje cells. Cortico-striatal overconnectivity could result from a loss of GABAergic projection neurons within the striatum. For instance, studies on Huntington’s disease showed loss of striatal projection neurons in the indirect pathway, and this is thought to tip the balance in favor of the direct pathway whose role is excitatory on cortex and hence cause abnormal involuntary movements (Reiner et al. 1988; Albin et al. 1992). Thus, we can speculate that GABAergic dysfunction may also underlie cortico-striatal overconnections reported in the presence of DCD. Localized proton magnetic resonance spectroscopy, which measures in vivo neurochemical information and has already been used in ASD children to show reduced GABA concentration (e.g., Gaetz et al. 2014; Rojas et al. 2014), may be useful to test this hypothesis and uncover the neurobiology of DCD.

A point which is worth mentioning is that default-mode cortico-cerebellar connectivity map contributed importantly to the decision function in all MKL models (TD-COM, TD-[DCD-COM], and TD-[DD-COM]). In particular, the results suggested atypical cerebellar connectivity targeting mainly the parieto-occipital sulcus in DD and/or DCD children. To our knowledge, this result is without precedent in DCD while there are studies that already reported disrupted connectivity with occipito-temporal areas in dyslexia (Shaywitz et al. 2003; van der Mark et al. 2011; Finn et al. 2014; Schurz et al., 2015). The parieto-occipital sulcus is known to be involved in tasks requiring visuo-spatial attention and working memory (Richter et al. 2019), domains where both DD and DCD children demonstrate deficits (Valdois et al. 2004; Ahissar 2007; Biotteau et al. 2017; Wilson et al. 2017). Hence, atypical cerebellar functional connectivity with the posterior occipito-temporal sulcus might be a defining feature of both diseases. In addition, MKL also identified particularities in cerebellar functional connectivity targeting the superior parietal lobule (i.e., dorsal attention cortico-cerebellar connectivity) as part of the DCD phenotype. Given that the dorsal attention network enables top–down cognitive selection of stimuli and actions (Corbetta et al. 2008), this deficit may relate to difficulties in executive function reported in DCD (Wilson et al. 2017). But, again, these are secondary outcomes that did not survive the consensus analysis. As such, they should only be considered as avenues for future research.

Finally, although our findings support the notion of a deficit in cortico-subcortical circuits that applies to DCD but not to DD, some limitations should be mentioned. First, our conclusion has been obtained in an indirect way with regards to MKL, mixing COM children with either DCD or DD children. Using it, we demonstrated that TYP vs [DCD-COM] model led to the same classification and kernels as TYP vs COM model, while accuracy decreased and some kernels get lost for TYP vs [DD-COM] model. Hence, the addition of DD children decreased the distance from COM children and brought the diseased children closer to the TYP children. We had to go through these posthoc MKL models to interpret TYP vs COM results given that direct classification of TYP vs DD or DCD did not reach significance (likely because of the low sample size for DCD). Of course, one has to keep in mind that our overall interpretation has also been established based on the GLM that identified atypical cortico-subcortical connectivity profiles in DCD and COM children compared with DD and TYP children. Another limit has to do with our parcellation strategy, which involved template-based parcels and seed-to-voxels methododology. Using this strategy, we were not able to reproduce the entire set of cortico-cerebellar and cortico-striatal networks reported in the original studies in adults (Buckner et al. 2011; Choi et al. 2012). Although the estimated cortico-cerebellar networks corresponded well to the adults seven networks, with the exception of an overlap between the somatomotor and ventral attention networks, the estimated cortico-striatal networks included only the sensorimotor network and incomplete default-mode and frontoparietal control networks. Hence, our template-based parcellation strategy have ‘missed’ some cortico-subcortical, especially cortico-striatal, functional networks. This may be because the segregation of these circuits is still underway by age 8–12 years, so that templates derived from adults were suboptimal. There is evidence that cortico-cerebellar and cortico-striatal functional connectivity is still far from having reached maturity by end childhood (Supekar et al. 2009; Cerliani et al. 2015; Cignetti et al. 2017; van Duijvenvoorde et al. 2019). Accordingly, it cannot be excluded that DD and/or DCD children may show abnormalities in other functional connectivity pathways than those revealed here. Some of the cortico-cerebellar networks may have also been slightly biased because of the seed-to-voxel approach. It has been documented blurring of fMRI signal across the cerebellar–cerebral boundary that leads especially to artifactual correlations between seeds spanning the cerebellum and the occipital cortex (Buckner et al. 2011). We believe that such a blurring issue may be responsible of the unexpected presence of occipital regions in our estimated cortico-cerebellar networks (Figs 1 and S1). Fortunately, group differences were not located in these regions, thus allowing us to exclude the possibility that our main findings may be biased. Beyond these methodological elements, we would like to stress that our study provides the first piece of evidence that fine parcellation of the cerebellum and of the striatum into multiple networks is necessary to address the etiology behind neurodevelopmental disorders, calling for more research on this topic. In this vein, an important next step will be to also evaluate whether circuit impairments such as those identified in the present study during resting state also manifest during task state.

## Conclusion

The results presented here indicate that functional somatomotor cortico-cerebellar and frontoparietal cortico-striatal circuits are impaired in DCD. This is compatible with current complementary explanatory frameworks of DCD, including procedural motor skill learning deficit and internal modelling deficit, thus providing them with a neural basis. On the other hand, the results run against the idea of an impaired cortico-subcortical connectivity in DD, and consequently the idea that the above frameworks are useful to conceptualize DD. As an alternative solution, we can allude speculatively the most popular phonological deficit framework of DD that is related to the left cerebral reading network. Just as important, the fact that DD-DCD comorbidity did not lead to more severely impaired circuits compared with DCD alone also casts doubts on a common brain origin to both disorders. Further studies are required to complete the picture of the boundaries and overlaps of brain systems subtending neurodevelopmental disorders. These studies should not be limited to DD and DCD but should also include other disorders (e.g., ASD, attention-deficit/hyperactivity disorder) to evaluate whether diagnostic categories of the DSM-5 represent homogeneous disorders or conversely whether they are intertwined and share a common etiology.

## Notes

*Conflict of Interest*: None declared.

## Funding

French National Research Agency (ANR-13-APPR-0010). F.C., M.V., and C.A. were also partially supported by the French National Centre for Space Studies.

## Supplementary Material

suppl_material_Cignetti_31_03_2020_CerebCortexComm_tgaa011
