# Supplementary material for: Intrinsic Cortico-Subcortical Functional Connectivity in Developmental Dyslexia and Developmental Coordination Disorder
Source: Cereb Cortex Commun. 2020 Apr 6;1(1):tgaa011. doi: 10.1093/texcom/tgaa011 (PMC8152893; doi:10.1093/texcom/tgaa011)
Supplement: suppl_material_Cignetti_31_03_2020_CerebCortexComm_tgaa011 [file suppl_material_cignetti_31_03_2020_cerebcortexcomm_tgaa011.docx]

**Supplementary data**

**
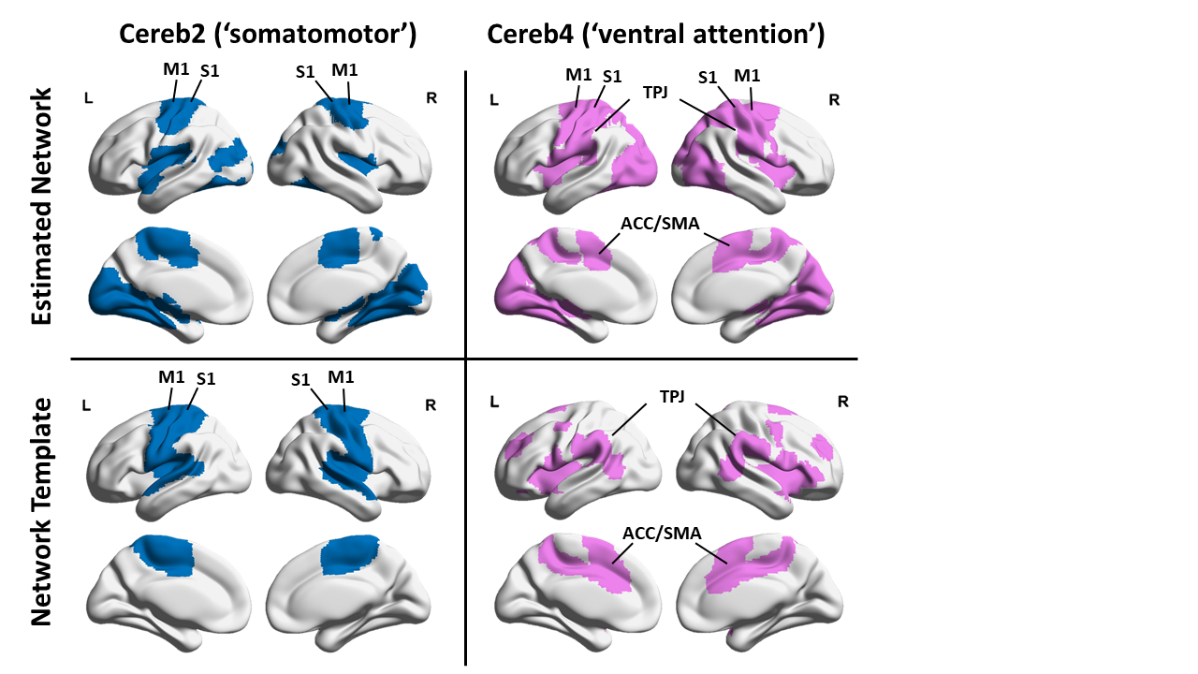
**

**Figure S1.** Somatomotor and ventral attention cortico-cerebellar functional connectivity networks. The estimated ventral attention cortico-cerebellar network was not limited to temporo-parietal and ventral frontal regions as the template network but also included motor and somatosensory cortical regions. It may also be noticed that both estimated networks included occipital regions, which might be more related to blurring of fMRI signal across the cerebellar-cerebral boundary (see discussion in that respect).

*Abbreviations: M1: primary motor cortex; S1: primary somatosensory cortex; SMA: supplementary motor area; ACC: anterior cingulate cortex; TPJ: temporo-parietal junction.*

**
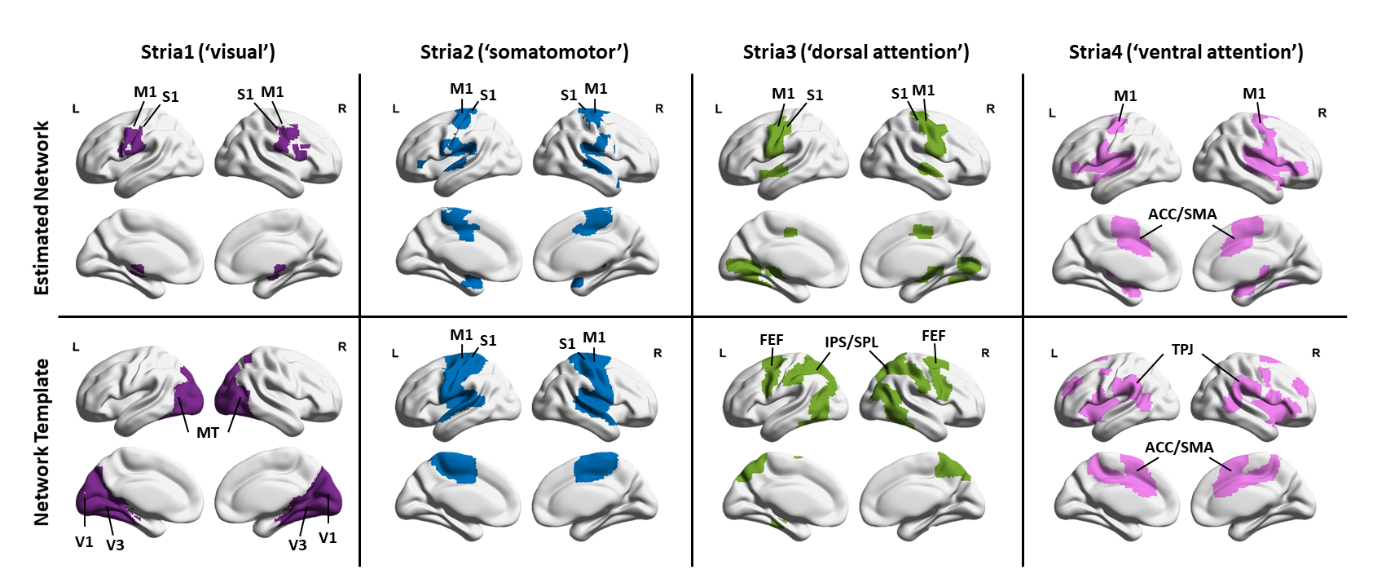
**

**Figure S2.** Visual, somatomotor, dorsal attention, and ventral attention cortico-striatal functional connectivity networks. There was a good topographic correspondence between estimated and template somatomotor networks. Inversely, all other estimated networks topographically differed from the template networks, involving connections established with sensorimotor regions. Accordingly, all these networks were labelled ‘somatomotor’ networks.

*Abbreviations: M1: primary motor cortex; S1: primary somatosensory cortex; SMA: supplementary motor area; ACC: anterior cingulate cortex; FEF: frontal eye field; IPS: intraparietal sulcus; SPL: superior parietal lobule; TPJ: temporo-parietal junction; MT: medial temporal area; V1: primary visual cortex; V3: visual area 3.*
